# Supplementary material for: Mitochondrial Reactive Oxygen Species in TRIF-Dependent Toll-like Receptor 3 Signaling in Bronchial Epithelial Cells against Viral Infection
Source: Int J Mol Sci. 2023 Dec 22;25(1):226. doi: 10.3390/ijms25010226 (PMC10778811; doi:10.3390/ijms25010226)
Supplement: Supplementary file 1 [file ijms-25-00226-s001.zip › Supplementary Materials.pdf]

Article

Mitochondrial Reactive Oxygen Species in TRIF-Dependent Toll-like Receptor 3 Signaling in Bronchial Epithelial Cells against Viral Infection

Ga Eul Chu [1,†], Jun Young Park [2,†], Chan Ho Park [1] and Won Gil Cho [1,*]

1 Department of Anatomy, Yonsei University Wonju College of Medicine 20, Ilsan-ro, Wonju 26426, Republic of Korea; xiahgaeul@naver.com (G.E.C.); wktlr8@naver.com (C.H.P.)

2 Department of Nuclear Medicine, Severance Hospital, Yonsei University College of Medicine,
50-1 Yonsei-ro, Seodaemun-gu, Seoul 03722, Republic of Korea; abies60@naver.com

* Correspondence: wch01@yonsei.ac.kr; Tel.: +82-33-741-0275

† These authors contributed equally to this work.

Supplementary material

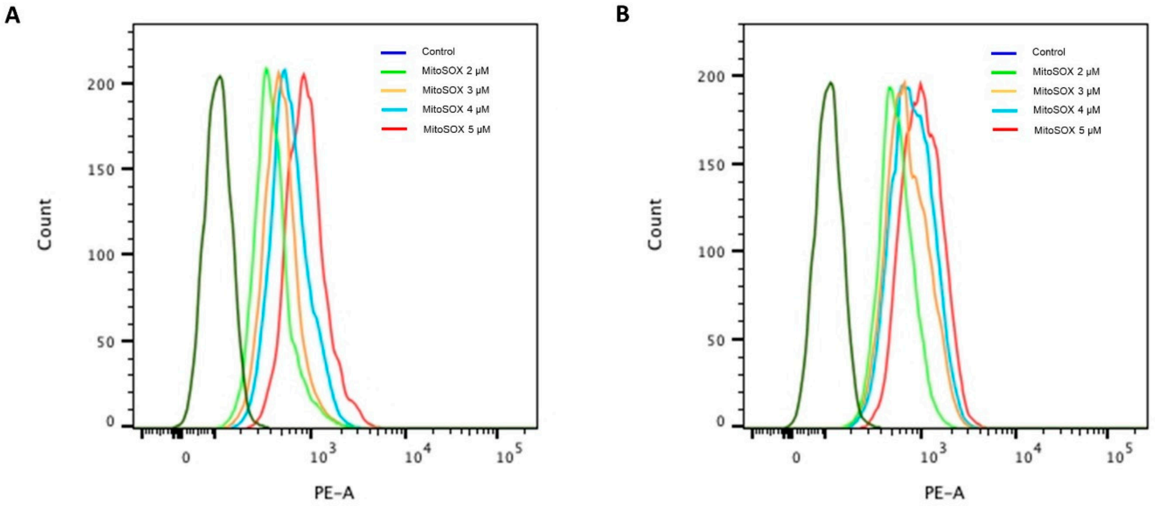

Supplemental Figure S1. The optimization of MitoSOX final concentration in bronchial epithelial cells. The analysis was conducted by flow cytometry at different concentrations. BEAS-2B cells were treated 2 μM to 5 μM of MitoSOX for 20 min dissolved in Hank's balanced salt solution (HBSS) and washed with DPBS. Cell were detached and transferred to FACS tube after resuspension with DPBS. Sample were analyzed by flow cytometry on an LSR II Flow Cytometer (Becton Dickinson, Franklin Lakes, NJ, USA) Data were analyzed using FlowJo software (Version 10.6.2, Tree Star Software. (**A,B**) BEAS-2B cells were stained with MitoSOX in a dose-dependent manner.

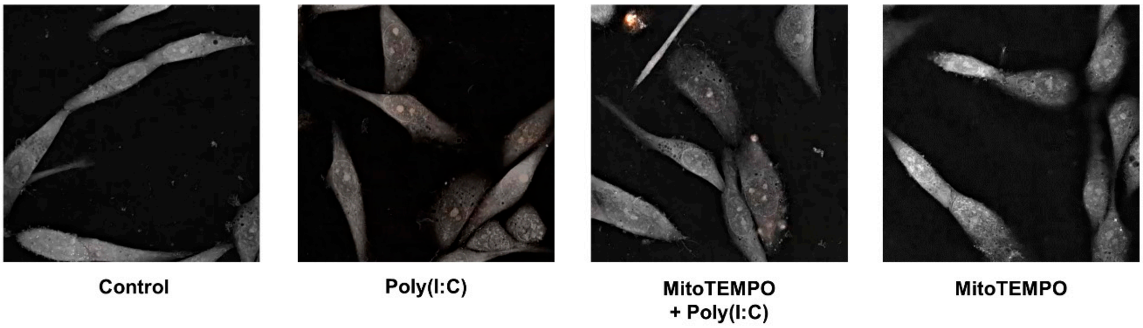

Supplemental Video S1-4. Live cell imaging of mtROS expression in bronchial epithelial cells. Live cell imaging was conducted in real-time using 3D Cell Explorer microscope. mtROS levels of BEAS-2B cells were determined by 2 μM Mito-SOX staining without 10 μg/mL poly(I:C) stimulation. Plates were replaced in 3D Cell Explorer microscope microscope (Nanolive, Ecublens, Switzerland) equipped with a top-stage incubator (Okolab S.R.L., Pozzuoli, Italy). The cells were observed for 6 h 37°C in 5% CO_2. Obtained images were processed using the Steve software v1.6.3496 (Nanolive).

Supplemental Video S2. BEAS-2B cells were treated 10 μg/mL poly(I:C) for 6h, and mtROS levels were determined by 2 μM Mito-SOX staining using 3D Cell Explorer microscope.

Supplemental Video S3. BEAS-2B cells were pre-treated 100 μM Mito-TEMPO for 1 h before imaging, and mtROS levels were determined by 2 μM Mito-SOX staining with 10 μg/mL poly(I:C) stimulation for 6 h using 3D Cell Explorer microscope.

Supplemental Video S4. BEAS-2B cells were pre-treated 100 μM Mito-TEMPO for 1 h before imaging, and mtROS levels were determined by 2 μM Mito-SOX staining without poly(I:C) stimulation using 3D Cell Explorer microscope.

Supplemental Table S1. CT values of real-time quantitative PCR

	**18S**			**TNF-α**					
	**CT1**	**CT2**	**Average CT**	**CT1**	**CT2**	**Average CT**	ΔCT	ΔΔCT	$2^{-\Delta\Delta CT}$
No treatment	14.35	14.45	14.40	34.71	34.31	34.51	34.31	1.00	0.25
Poly(I:C)	14.22	14.13	14.17	27.89	27.72	27.81	27.72	9.41	1.07
Poly(I:C)+ TLR3 siRNA	14.27	14.54	14.41	33.19	32.22	32.70	32.22	3.16	0.30
Poly(I:C)+ TLR3 siRNA+ MitoTEMPO	13.55	13.69	13.62	30.27	30.22	30.25	30.22	2.34	0.47
TLR3 siRNA	14.35	14.37	14.36	33.93	33.14	33.54	33.14	1.03	0.27
MitoTEMPO	15.82	16.19	16.00	34.41	33.34	33.87	33.34	1.20	0.49

	**18S**			**IL-16**					
	**CT1**	**CT2**	**Average CT**	**CT1**	**CT2**	**Average CT**	ΔCT	ΔΔCT	$2^{-\Delta\Delta CT}$
No treatment	14.35	14.45	14.40	34.23	34.66	34.45	34.66	1.00	0.63
Poly(I:C)	14.22	14.13	14.17	26.44	26.54	26.49	26.54	246.14	16.15
Poly(I:C)+ TLR3 siRNA	14.27	14.54	14.41	34.69	33.50	34.10	33.50	110.37	13.25
Poly(I:C)+ TLR3 siRNA+ MitoTEMPO	13.55	13.69	13.62	26.07	26.20	26.13	26.20	16.61	5.11
TLR3 siRNA	14.35	14.37	14.36	33.65	33.76	33.70	33.76	1.13	0.78
MitoTEMPO	16.05	16.23	16.19	32.09	32.10	32.10	32.10	1.71	0.56
